# A Video Interview With Richard J. Warren, MD: My Second Thoughts About the High Superficial Musculoaponeurotic System

**DOI:** 10.1093/asjof/ojac093

**Published:** 2022-12-21

**Authors:** Richard J Warren, Lorne King Rosenfield

**Affiliations:** private practice, Vancouver, Canada; Department of Plastic Surgery, University of California San Francisco, San Francisco, CA, USA

Welcome to another *Second Thoughts on First Thoughts* offering. Today, we interview one of our most respected colleagues from the Great White North: Dr Richard J. Warren (Video). His learning turning point was shifting from a high superficial musculoaponeurotic system (SMAS) strategy to a lower SMASectomy à la Baker. The interview follows the format of the 5 Ws in investigative reporting: Who, What, Where, When, and Why? in honor of the longest-running Canadian TV show of its kind, “W5.” We invite you to listen in on our conversation to learn the answers to those same questions!

## Supplementary Material

ojac093_Supplementary_Data

